# Supplementary material for: Compact high power, medium energy electron accelerator for treatment of per- and polyfluoroalkyl contamination in water
Source: PLoS One. 2026 Jan 14;21(1):e0323581. doi: 10.1371/journal.pone.0323581 (PMC12803471; doi:10.1371/journal.pone.0323581)
Supplement: Attachment — Submitted filename: Response to Reviewers.pdf [file pone.0323581.s002.pdf]

Compact High Power, Medium Energy Electron Accelerator for Treatment of Per- and

Polyfluoroalkyl Contaminations in Water - EBWT@SEALAB

Dear Mr. Bonatto and dear Reviewer,

We thank you for the constructive and detailed feedback on our manuscript *"Compact High Power, Medium Energy Electron Accelerator for Treatment of Per- and Polyfluoroalkyl Contaminations in Water – EBWT@SEALab"*. Your comments have helped us improve the clarity, accuracy, and completeness of our work. Below, we respond point-by-point to all suggestions and questions. Reviewer and editor comments are presented in *italics*, our responses in plain text. All line numbers refer to the revised manuscript.

General comments:

*The current study aims to investigate a proof-of-concept experiment of a new in-air beamline electron beam system to potentially degrade PFAS in aqueous and theoretically other matrices. It is extremely important to investigate innovative technologies to completely degrade PFAS and e-beam technology is one such approach. The authors have done a good job of summarizing existing literature on this field and accordingly comparing their proof-of concept device to these devices. This information is very relevant and is necessary to the PFAS community. This paper can be improved with more explanation and discussion of certain points, as suggested below. I recommend "**Major revisions**" to the manuscript at this stage.*

We addressed the "Major Revisions" by systematically reviewing the manuscript for technical consistency, improving the cost analysis, refining explanations, and enhancing readability. Content changes were made where requested, and additional clarifications were added to strengthen the manuscript

Comments by line:

Line 16: *Does not require citation, it is commonly known that it is a human right.*

We removed the citation as suggested.

Lines 25-27: *As a suggestion, please add a few sentences describing the current treatment technologies to remove and destroy PFAS and provide a more detailed pros and cons list of each of these technologies.*

Lines 30–40 now describe granular activated carbon (GAC), ion exchange (IX), and reverse osmosis (RO) methods, with advantages (scalability, regulatory acceptance) and disadvantages (secondary waste generation, reduced efficiency for short-chain PFAS). Lines 40–45 contrast these with EBWT, highlighting its destructive, additive-free nature.

Lines 52-53: *Which key PFAS? Please specify the PFAS and provide a list/grouping of the 20 PFAS mentioned.*

Lines 66–71 now define PFAS-4 (PFOA, PFNA, PFHxS, PFOS) and PFAS-20 (20 specific C4–C13 perfluorocarboxylic and perfluorosulfonic acids) as per the EU Drinking Water Directive.

Line 61: *Highly contaminated means what concentrations?*

Lines 73–78 now explain that treated water is reintroduced into the system and filters are replaced about twice per year. Additional context (lines 78–86) contrasts our EBWT vision with current filtration methods.

Line 80, 130: *Formatting error, please correct citation.*

All formatting errors were corrected.

Line 103: *Check units for the cost.*

We reviewed all cost units and corrected inconsistencies throughout the manuscript. The cost analysis was expanded (lines 113–116, 130–132, 144–146, 150–151, 161–166) to detail accelerator types, capacities, and operating expenses.

Line 127: *Define first usage- HZB, FWHM.*

First mentions now include full definitions: Helmholtz-Zentrum Berlin (HZB) and full width at half maximum (FWHM).

Lines 191-195: *Is it well accepted that higher dose rates means higher abundance of hydrated electrons? Is there a cutoff after which scavenging reactions dominate formation of hydrated electrons and their relative abundance?*

Lines 244–248 now explain that higher dose rates do not necessarily yield more hydrated electrons due to scavenging species. Our study aims to identify the optimal dose rate.

Lines 212-213: *Please reframe this statement, "to achieve a minimum dose of XX kGY".*

Line 267 now specifies a minimum target dose of 10 kGy based on prior successful studies.

Lines 234-235: *So how are the authors determining that the dose is high enough to degrade PFAS? Is this based on previous studies that have looked at PFAS degradation? Or is this based on model output? Does this actually consider the interactions between PFAS (C-F bonds) and hydrated electrons?*

Lines 291–299 explain that our dose assumptions are based on previous studies achieving PFAS degradation in similar dose ranges. The planned experiment will verify this in practice.

Based on the operational parameters, what is the range of e-beam dose that can be delivered, and can the dose rate be tweaked?

Lines 436–439 state that our system can deliver dose rates up to several kGy/s; final average dose depends on setup parameters, which can be tuned experimentally.

Additional comments by the Editor

1. Please ensure that your manuscript meets PLOS ONE's style requirements, including those for file naming. The PLOS ONE style templates can be found at https://journals.plos.org/plosone/s/file?id=wjVg/PLOSOne_formatting_sample_main_body.pdf and https://journals.plos.org/plosone/s/file?id=ba62/PLOSOne_formatting_sample_title_authors_affiliations.pdf

Double spacing was adjusted, the Fig7 was named correctly in the file upload. The affiliations were all corrected.

2. Please note that PLOS ONE has specific guidelines on code sharing for submissions in which author-generated code underpins the findings in the manuscript. In these cases, we expect all

author-generated code to be made available without restrictions upon publication of the work. Please review our guidelines at https://journals.plos.org/plosone/s/materials-and-software-sharing#loc-sharing-code and ensure that your code is shared in a way that follows best practice and facilitates reproducibility and reuse.

We have deposited all author-generated code underpinning our results in Zenodo, ensuring public access without restrictions. The repository contains all scripts, documentation, and example data needed to reproduce our analyses. It is archived under the MIT License, in line with PLOS ONE's code-sharing guidelines. DOI: https://doi.org/10.5281/zenodo.16894583

3. Please amend your list of authors on the manuscript to ensure that each author is linked to an affiliation. Authors' affiliations should reflect the institution where the work was done (if authors moved subsequently, you can also list the new affiliation stating "current affiliation:…." as necessary).'

We corrected the author list to ensure each author is linked to the correct affiliation at the time of the work.

4. Please ensure that you refer to Figure 7 in your text as, if accepted, production will need this reference to link the reader to the figure.

We now explicitly reference Figure 7 in the main text

5. We note you have included a table to which you do not refer in the text of your manuscript. Please ensure that you refer to Table 3, 4, and 5. in your text; if accepted, production will need this reference to link the reader to the Table.

Tables 3, 4, and 5 are now cited in the manuscript to ensure proper linking during production.

We sincerely thank Mr. Bonatto and the Reviewer for their valuable feedback, which has improved the clarity, technical accuracy, and overall quality of our manuscript. We believe the revisions fully address all comments and hope the revised version meets the expectations for acceptance.

Sincerely,

Tasha Spohr
