# Supplementary material for: Compact high power, medium energy electron accelerator for treatment of per- and polyfluoroalkyl contamination in water
Source: PLoS One. 2026 Jan 14;21(1):e0323581. doi: 10.1371/journal.pone.0323581 (PMC12803471; doi:10.1371/journal.pone.0323581)
Supplement: Attachment — Submitted filename: PONE-D-25-19239 - Reviewer.docx [file pone.0323581.s001.docx]

**Compact High Power, Medium Energy Electron Accelerator for Treatment of Per- and**

**Polyfluoroalkyl Contaminations in Water - EBWT@SEALAB**

**General comments:** The current study aims to investigate a proof-of-concept experiment of a new in-air beamline electron beam system to potentially degrade PFAS in aqueous and theoretically other matrices. It is extremely important to investigate innovative technologies to completely degrade PFAS and e-beam technology is one such approach. The authors have done a good job of summarizing existing literature on this field and accordingly comparing their proof-of concept device to these devices. This information is very relevant and is necessary to the PFAS community. This paper can be improved with more explanation and discussion of certain points, as suggested below. I recommend “**Major revisions**” to the manuscript at this stage.

**Comments by line:**

**Line 16:** Does not require citation, it is commonly known that it is a human right

**Lines 25-27:** As a suggestion, please add a few sentences describing the current treatment technologies to remove and destroy PFAS and provide a more detailed pros and cons list of each of these technologies

**Lines 52-53:** Which key PFAS? Please specify the PFAS and provide a list/grouping of the 20 PFAS mentioned

**Line 61:** Highly contaminated means what concentrations?

**Line 80, 130:** Formatting error, please correct citation

**Line 103:** Check units for the cost

**Line 127:** Define first usage- HZB, FWHM

**Lines 191-195:** Is it well accepted that higher dose rates means higher abundance of hydrated electrons? Is there a cutoff after which scavenging reactions dominate formation of hydrated electrons and their relative abundance?

**Lines 212-213:** Please reframe this statement, “to achieve a minimum dose of XX kGY"

**Lines 234-235:** So how are the authors determining that the dose is high enough to degrade PFAS? Is this based on previous studies that have looked at PFAS degradation? Or is this based on model output? Does this actually consider the interactions between PFAS (C-F bonds) and hydrated electrons?

Based on the operational parameters, what is the range of e-beam dose that can be delivered, and can the dose rate be tweaked?
